# Social Support and Spiritual Well-Being of Patients With Esophageal Cancer Aged Over 50 Years: The Mediating Role of Rumination

**DOI:** 10.3389/fpsyt.2022.805380

**Published:** 2022-03-04

**Authors:** Jingran Li, Liang Xue, Hailong Pan

**Affiliations:** ^1^School of Nursing, Yangzhou University, Yangzhou, China; ^2^Department of Nursing, Affiliated Hospital of Yangzhou University, Yangzhou, China

**Keywords:** esophageal cancer, spiritual well-being, social support, deliberate rumination, intrusive rumination

## Abstract

**Background:**

Spiritual well-being plays an important role in helping patients cope with disease. Previous studies have investigated the association between social support and spiritual well-being, whereas few studies have explored the relationship in patients with esophageal cancer (EC), and the mechanisms behind this pathway have not been thoroughly examined.

**Objective:**

This study aimed to explore the relationship between social support and spiritual well-being of Chinese patients with EC aged over 50 years and to analyze whether the relationship was mediated by rumination.

**Methods:**

A cross-sectional survey was conducted with 197 EC patients. Participants completed the general information questionnaire, the Functional Assessment of Chronic Illness Therapy–Spiritual Scale, the Chinese Event Related Rumination Inventory, and the Perceived Social Support Scale (PSSS).

**Results:**

Results demonstrated that social support of patients with EC aged over 50 years was positively correlated with spiritual well-being and deliberate rumination and negatively correlated with intrusive rumination; spiritual well-being was positively associated with deliberate rumination and negatively correlated with intrusive rumination. The effect of social support on spiritual well-being was partially mediated by deliberate rumination and intrusive rumination.

**Conclusions:**

The findings suggest that interventions directed toward enhancing social support and deliberate rumination and reducing the level of intrusive rumination may help patients with EC aged over 50 years improve spiritual well-being.

## Introduction

Esophageal cancer (EC) is a malignant disease that impacts the Chinese population. More than 50% of new cases of EC occur in China (1). In China, EC ranks sixth in incidence and fourth in mortality, both higher than the world rankings (2). It has become one of the common causes of cancer-related death in China (3). EC mainly occurs in middle-aged and elderly individuals, and because of the aging population, the incidence in older adults will continue to rise (4). EC has no obvious symptoms in the early stages, and most patients are already in the middle or late stages when their disease is diagnosed. Because of poor prognosis, most patients with EC suffer from psychological problems such as anxiety and depression (5). Simultaneously, these emotional changes have a negative effect on their spiritual well-being.

Spiritual well-being is an outcome indicator of an individual's ability to cope with threats and changes throughout their lifespan (6). It is considered to be a basic dimension of overall human health (7), which is embodied in four aspects: the relationships with oneself, others, the environment, and/or transcendence (8). As a positive manifestation of an individual's internal coping, spiritual well-being is an unmatched power for coordinating our physical, psychological, and social dimensions (9). It plays a significant role in helping patients reduce negative emotions and reconstruct the meaning and purpose of life (10), and it is greatly important to the survival and development of human society. Studies have also indicated that spiritual health, as a resource to support adaptation, plays a vital role in helping individuals to cope with disease (11). In a longitudinal study across a span of more than 60 years, scholars showed that levels of spirituality increased obviously, especially from middle to late adulthood (12). In this regard, studying positive constructs in older patients may be helpful to understand their development throughout their lifespan. Therefore, our research aimed to investigate the spiritual well-being of patients with EC older than 50 years and to identify its influencing factors.

Social support refers to an action that gives assistance to others through certain mental or material means to help them cope with stress (13). As a positive external source, social support helps patients relieve psychological distress and improve their quality of life (14, 15). Some scholars have indicated that social support plays a critical part in cancer patients' spiritual well-being. For example, Ciria-Suarez et al. (16) tested the correlation between social support and spiritual health in cancer patients and found that social support was significantly correlated with spiritual well-being. Although social support may predict spiritual well-being, the mechanism behind this pathway has not been thoroughly examined. More importantly, few studies have explored the correlation between social support and spiritual health in Chinese patients with EC. Our study focused on a potential mediator between social support and spiritual well-being: rumination.

Rumination is defined as a form of repetitive negative thinking characterized by recurrent thoughts and self-focused attention (17, 18) on negative emotions and symptoms and their causes, meanings, and consequences (19). A recent study suggested that rumination, as a form of repetitive negative thinking, could be associated with several poor psychological outcomes in adulthood, for example, eating disorders (20), psychological disorders, and psychiatric symptoms (21). Kaplan et al. found that rumination could also lead to affective disorders (22). Moreover, rumination could be a useful cognitive strategy for shy individuals (23). Although rumination is generally correlated with negative clinical outcomes, as a cognitive process, it can also contribute to individual growth after stressful life events (24). In such a context, rumination is divided into two different forms: intrusive and deliberate (25). The former refers to the passive and repeated obsessive thinking of individuals after a traumatic event, and the latter refers to the active thinking of individuals about stressful events (26). Many scholars have demonstrated that deliberate rumination could help patients recover from the negative effects of stressful events, promote self-growth, and help them adapt to the disease (27). However, intrusive rumination makes patients focus on the negative influence of traumatic experiences, which increases their negative evaluation of those stressful events (18). In addition, higher intrusive rumination about suffering was related to poor well-being (28). However, it is not clear whether rumination can directly affect spiritual well-being. Moreover, regarding the association between social support and rumination, it was reported that higher social support tended to lead to lower intrusive rumination and higher deliberate rumination in Chinese adolescents (29). In addition, social support was considered an important factor that affected intrusive rumination and deliberate rumination among accidentally injured patients (30).

This study was guided by social–cognitive processing (SCP) theory (31). According to SCP theory, supportive social networks may enhance active cognitive processing of a trauma experience, thus leading to integration of trauma-related information and, ultimately, positive adaptation. However, an unsupportive social environment seems to potentiate intrusive rumination, which may lead to negative adaptation. In our study, social support was considered to be the antecedent factor of rumination, and spiritual well-being referred to the outcome after adaptation. Therefore, we hypothesized that rumination plays a mediating role between social support and spiritual well-being.

As discussed above, our study aimed to (1) analyze the association between social support, rumination, and spiritual well-being in Chinese patients with EC aged over 50 years and (2) explore whether rumination plays a mediating role between social support and spiritual well-being.

## Materials and Methods

### Recruitment and Participants

We utilized a descriptive, cross-sectional design. From December 2020 to June 2021, a total of 197 patients with EC aged over 50 years were recruited from two hospitals in China. Tumor-node-metastasis (TNM) staging and assessment of the depth of cancer invasion were based on the American Joint Committee on Cancer standards (32). Inclusion criteria of patients were as follows: (1) had been clinically diagnosed with EC in TNM stages II–IV; (2) ≥50 years old; (3) could fill out the questionnaire independently or with assistance; and (4) were aware of their condition. The exclusion criteria were as follows: (1) severe heart, lung, kidney, and other organ diseases; (2) cognitive function and mental disorders; (3) severe mental illness; and (4) declined to participate in the study.

To ensure the accuracy of the data, data collectors were trained. Then, we introduced the purpose of our study to the patients. Once we obtained verbal consent from the patients, the investigation was conducted. First, the patients were given informed consent forms to sign. Next, we gave them paper questionnaires and explained the requirements and precautions. After the patients filled out the questionnaires, the data collectors checked the questionnaires on the spot. For patients who could not read or fill in the questionnaire, we explained the content of the questionnaire to them in an objective tone and assisted them in filling it out. A total of 209 patients agreed to participate in this survey, and 197 patients finally completed the survey. The effective response rate of the questionnaire was 94.3%.

### Measures

#### Spiritual Well-Being

We used the Chinese Functional Assessment of Chronic Illness Therapy–Spiritual Scale (FACIT-Sp-12) to measure patients' spiritual well-being (33). The FACIT-Sp-12 is composed of 12 items, which fall into three dimensions: (1) belief, (2) meaning, and (3) sense of peace. All items were rated on a 4-point Likert scale (0 = not at all to 4 = very). The total score was calculated by summing all scale items (total FACIT-Sp-12 score ranges from 0 to 48). Patients with higher FACIT-Sp-12 scores had higher levels of spiritual well-being. The overall Cronbach α coefficient in the cancer patients was 0.831.

#### Rumination

The Chinese version of the Event-Related Rumination Inventory (34) was used to measure patients' rumination. The scale consists of 20 items and 2 subscales: deliberate rumination (10 items) and intrusive rumination (10 items), which represent the positive and negative aspects of patients' cognitive processing, respectively. All items were rated on a 4-point Likert scale (0 = never have such thoughts to 3 = always have such thoughts). The total scores of each dimension range from 0 to 30, and the total scores range from 0 to 60 points. A higher score indicated a higher level of rumination. The overall Cronbach α coefficient was 0.92, and the Cronbach α coefficients of the two subscales were 0.93 and 0.85, respectively.

#### Social Support

To measure social support, patients completed the Perceived Social Support Scale (PSSS), which was developed by Zimet et al. (35). The scale consists of 12 items and 3 dimensions: (1) family support, (2) friend support, and (3) other support. Each item is rated on a 7-point Likert scale (1 = strongly disagree to 7 = strongly agree). Total scores ranging from 12 to 36 indicated a low level of social support, total scores ranging from 37 to 60 revealed a moderate level of social support, and total scores ranging from 61 to 84 indicated a high level of social support. The Cronbach α coefficient of the scale was 0.85.

### Statistical Analysis

Data analysis was performed using IBM SPSS 26.0 software. Descriptive statistics were conducted to display the demographic and clinical characteristics. The scores of social support, deliberate rumination, intrusive rumination, and spiritual well-being were expressed as frequencies, means, and standard deviations. The Kolmogorov–Smirnov test indicated that the scores of social support, deliberate rumination, intrusive rumination, and spiritual well-being all conformed to a normal distribution. Therefore, Pearson correlation analysis was used to explore the relationship between all variables. Hierarchical multiple regression analysis was used to examine deliberate rumination and intrusive rumination as potential mediators of the association between social support and FACIT-Sp-12. The significance of the model was tested by bootstrap software (36). If the 95% confidence interval (CI) did not include 0, this would demonstrate significant mediation. The *p*-value was set at < 0.05.

## Results

### Sample Characteristics

Table 1 shows the demographic and clinical characteristics. The age of the patients ranged from 50 to 83 years (mean = 66.87 [SD = 7.42] years), and most of them were men (75.6%), married (82.7%), and had no religious belief (90.9%). In addition, 72.1% of participants were at stages III and IV of EC.

**Table 1:** Demographic and clinical characteristics of 197 participants (*N* = 197).

**Category**	**Group**	***n***	**%**
Gender	Male	149	75.6
	Female	48	24.4
Age	50–59 years	39	19.8
	60–69 years	94	47.7
	≥70 years	64	32.5
Education	Primary school or below	74	37.6
	Middle school	96	48.7
	High/secondary school	20	10.1
	Three-year college or above	7	3.6
Household monthly income	<3,000 RMB	72	36.6
	3,000–5,000 RMB	96	48.7
	>5,000 RMB	29	14.7
Marital status	Married	163	82.7
	Not partnered	34	17.3
Religious belief	Yes	18	9.1
	No	179	90.9
Personality type	Introverted	57	28.9
	Between introverted and outgoing	62	31.5
	Outgoing	78	39.6
Disease stage	Stage II	55	27.9
	Stage III	94	47.7
	Stage IV	48	24.4
Time since diagnosis	<6 months	91	46.2
	6–12 months	47	23.8
	1–3 years	37	18.8
	>3 years	22	11.2

### Correlations Among Spiritual Well-Being, Social Support, Deliberate Rumination, and Intrusive Rumination

The scores of social support, intrusive rumination, deliberate rumination, and spiritual well-being were 60.86 ± 8.45, 12.50 ± 5.09, 14.02 ± 4.53, and 26.50 ± 7.14, respectively. Table 2 shows the correlation for each variable. The Pearson correlation results showed that social support was positively related to deliberate rumination (*r* = 0.428, *p* < 0.001) and spiritual well-being (*r* = 0.497, *p* < 0.001), and negatively correlated with intrusive rumination (*r* = −0.367, *p* < 0.001). Deliberate rumination was positively associated with spiritual well-being (*r* = 0.521, *p* < 0.001), and intrusive rumination was negatively associated with spiritual well-being (*r* = −0.471, *p* < 0.001).

**Table 2:** Relationships between perceived social support, rumination, and spiritual well-being.

**Variables**	**Mean ±SD**	**1**	**2**	**3**	**4**
PSSS	60.86 ± 8.45	1			
Intrusive rumination	12.50 ± 5.09	−0.367^**^	1		
Deliberate rumination	14.02 ± 4.53	0.428^**^	−0.307^**^	1	
FACIT-Sp-12	26.50 ± 7.14	0.497^**^	−0.471^**^	0.521^**^	1

*PSSS, Perceived Social Support Scale; FACIT-Sp-12, Functional Assessment of Chronic Illness Therapy–Spiritual Scale.*

** *P < 0.01*.

### The Mediating Role of Rumination Between Social Support and Spiritual Well-Being

As seen in Table 3, in the first step, social support (independent variable) had a direct and positive predictive effect on spiritual well-being (outcome variable) without intrusive rumination and deliberate rumination (mediating variables) (β = 0.497, *t* = 7.996, *p* < 0.001). In the second step, social support was the independent variable, and intrusive rumination and deliberate rumination were the outcome variables. Social support had a negative predictive effect on intrusive rumination (β = −0.367, *t* = −5.508, *p* < 0.001) and a positive predictive effect on deliberate rumination (β = 0.428, *t* = 6.604, *p* < 0.001). In the third step, social support, intrusive rumination, and deliberate rumination were independent variables, and spiritual well-being was the outcome variable. Intrusive rumination (β = −0.277, *t* = −4.651, *p* < 0.001) and deliberate rumination (β = 0.327, *t* = 5.346, *p* < 0.001) were introduced into the regression equation as mediating variables that affected the positive predictive effect of social support on spiritual well-being (β = 0.255, *t* = 4.079, *p* < 0.001).

**Table 3:** The mediating role of intrusive rumination and deliberate rumination between perceived social support and spiritual well-being.

**Step**	**Outcome variable**	**Independent variable**	**β**	***F***	***R* ^2^**	***Adjusted R* ^2^**
First	FACIT-Sp-12	PSSS	0.497^**^	63.928	0.247	0.243
Second	Intrusive rumination	PSSS	−0.367^**^	30.338	0.135	0.130
	Deliberate rumination		0.428^**^	43.609	0.183	0.179
Third	FACIT-Sp-12	PSSS	0.255^**^	48.101	0.428	0.418
		Intrusive rumination	−0.277^**^			
		Deliberate rumination	0.327^**^			

*PSSS, Perceived Social Support Scale; FACIT-Sp-12, Functional Assessment of Chronic Illness Therapy–Spiritual Scale.*

** *P < 0.001*.

**Figure 1:**
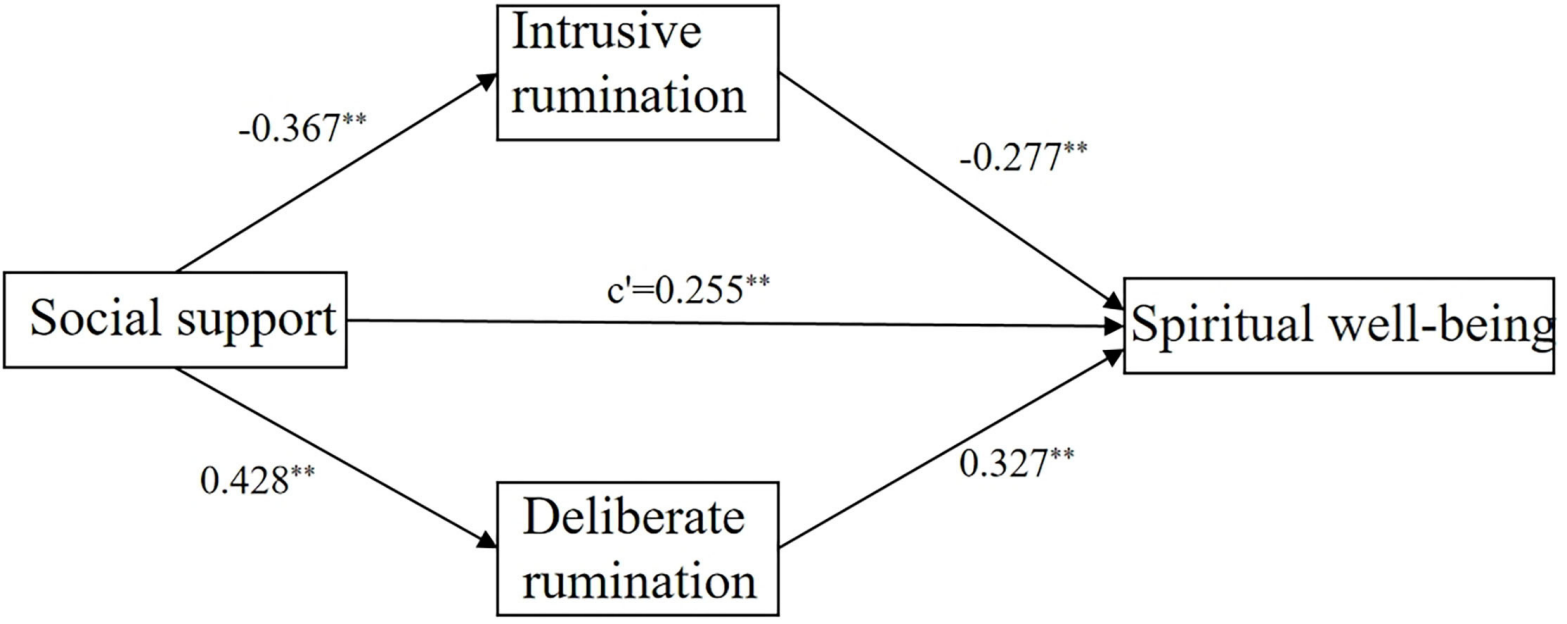
Structural routes of social support, intrusive rumination, deliberate rumination, and spiritual well-being among 197 Chinese patients with esophageal cancer aged over 50 years. **P < 0.001.

Bootstrap software (setting 2,000 repeated samplings and 95% CI) was used to examine the mediation effect of the model. The model diagram of the relationship between social support, intrusive rumination, deliberate rumination, and spiritual well-being is shown in Figure 1. As seen in Table 4, the direct effect of social support on spiritual well-being was 0.255, the total effect was 0.497, and the indirect effect was 0.242, accounting for 48.7% of the total effect; the mediating effect of intrusive rumination was 0.102, accounting for 20.5% [(−0.277) × (−0.367)/0.497 × 100%] of the total effect, and the mediating effect of deliberate rumination was 0.140, accounting for 28.2% [0.327 × 0.428/0.497 × 100%] of the total effect. Because zero was not included in the 95% CI, the mediating effect of each path was significant.

**Table 4:** The results of the mediation analysis.

**Path**	**Coefficient**	**Boot SE**	**95% CI**	
			**Lower**	**Upper**
Total path (PSSS → FACIT-Sp-12)	0.497	0.062	0.374	0.619
Direct path (PSSS → FACIT-Sp-12)	0.255	0.063	0.132	0.379
Indirect path	0.242	0.052	0.150	0.355
PSSS → deliberate rumination → FACIT-Sp-12	0.140	0.035	0.080	0.216
PSSS → intrusive rumination → FACIT-Sp-12	0.102	0.042	0.037	0.201

*PSSS, Perceived Social Support Scale; FACIT-Sp-12, Functional Assessment of Chronic Illness Therapy–Spiritual Scale; CI, confidence interval*.

## Discussion

As an important element for overall health, spirituality has been considered a universal trait by which patients look for meaning in their life (37), and has been demonstrated to be positively correlated with quality of life (38). Among the participants in our study, only 9.1% had a high level of spiritual well-being, and the mean score of the FACIT-Sp-12 was 26.50 (SD = 7.14), which was lower than that reported by Zhang et al. (39). The lower scores can be explained by two factors. First, early clinical symptoms of EC are not obvious, and most patients are diagnosed at an advanced stage (40). Compared with other diseases, EC has a poor prognosis (41), and physiological functions are severely impaired, meaning patients face psychological problems such as anxiety, depression, fear, and the loss of meaning and spirituality in life (42). Second, most participants with lower education levels cannot properly develop an understanding of the disease (43) and seek inner peace. The aforementioned factors together lead to poor spiritual well-being. Therefore, it is essential to confirm the influencing factors and develop a proposal to improve patients' spiritual well-being.

SCP theory was put to good use in this study, which found that patients with EC who are in a supportive social environment experience fewer intrusive thoughts and more deliberate rumination, thus promoting spiritual well-being.

Social support has been considered to have a significant protective influence on mental health (44) and spiritual health (45). Good social support is an important spiritual force for cancer patients, allowing them to feel care and connection and realize the meaning and value of life (46). In this study, we found that there was a direct positive correlation between social support and spiritual well-being. Patients with EC aged over 50 years had higher FACIT-Sp-12 scores when they received more social support. Fombuena et al. (47) found that social support positively predicted spiritual well-being in cancer patients. In the study by Qi et al. (48), social support had a significant impact on spiritual well-being. In addition, social support was also demonstrated to be positively correlated with spiritual well-being for burn survivors (49). Therefore, intervention programs involving social support should be adapted for Chinese patients with EC aged over 50 years to improve their spiritual well-being.

Meanwhile, our findings indicated that the correlation between social support and spiritual health was mediated by intrusive rumination. The significant indirect effect suggested that rumination-targeted intervention would be beneficial for improving Chinese EC patients' spiritual well-being. Social support can provide individuals with resources to cope with traumatic events, which can make it easier for patients to eliminate trauma-relayed cues. A previous study (50) showed that patients with lower social support were more likely to experience intrusive rumination. Intrusive rumination is considered as a negative coping style, which makes it difficult for patients to find positive meaning in trauma events (51), further affecting their spiritual well-being.

Furthermore, we also found that deliberate rumination mediated the effect of social support on spiritual well-being among Chinese patients with EC aged over 50 years. Our findings demonstrated that social support could not only directly increase EC patients' spiritual well-being but also be effective through increasing patients' deliberate rumination. One possible interpretation was that social support can integrate the meaning of traumatic events and reduce the negative impact of traumatic events on individuals (52). Patients who receive more social support are more likely to adopt proactive coping strategies to promote their psychological adjustment and spiritual health and growth. Therefore, when explaining the impact of social support on spiritual well-being, we must emphasize the role of deliberate rumination. In addition, spiritual care intervention projects involving elements of psychological and social support should be developed to improve spiritual well-being.

This study helps us to better understand the association between social support, rumination, and spiritual well-being in Chinese patients with EC aged over 50 years. According to the results, improving the social support (e.g., their ability to use resources) of patients with EC aged over 50 years may reduce intrusive rumination and increase deliberate rumination, which ultimately enhances spiritual well-being. However, current medical interventions may ignore the important impact of social support. Furthermore, a number of psychological treatments have been developed to reduce rumination and were confirmed to be effective. For example, several studies have suggested that rumination-based therapy (53) or metacognitive therapy (54) should be considered potential protectors against intrusive rumination. Well-being therapy (55, 56) was also designed to enhance well-being in chronic diseases (57). Such interventions can help patients reconstruct their perception of stressful experiences and thus promote psychological growth.

However, our study had the following limitations. First, this was a cross-sectional study; longitudinal designs should be conducted in future research. Second, we only selected two hospitals in China for investigation, the scope was limited, and the sample size was small, so the results may be biased. Third, the level of social support, intrusive rumination, deliberate rumination, and spiritual well-being was measured with self-reported questionnaires in the present study; thus, inflation in the findings cannot be ignored because of subjective bias from patients. Fourth, early-stage EC patients were not included in this study, which may be an important reason for the lower level of spiritual well-being of patients with EC aged over 50 years. Finally, the current study mainly investigated the relationship between social support, intrusive rumination, deliberate rumination, and spiritual well-being, which led to us overlooking other factors influencing spiritual well-being.

## Conclusion

As we expected, intrusive rumination and deliberate rumination played a mediating role between social support and spiritual well-being. The findings highlighted the significance of potential interventions to reduce intrusive rumination and increase deliberate rumination and subsequently improve spiritual well-being of Chinese patients with EC aged over 50 years.

## Data Availability Statement

The raw data supporting the conclusions of this article will be made available by the authors, without undue reservation.

## Ethics Statement

The studies involving human participants were reviewed and approved by the Ethics Committee of Yangzhou University (No. YZUHL2021012). The patients/participants provided their written informed consent to participate in this study.

## Author Contributions

LX: concept and design. JL: acquisition, analysis or interpretation of data, and drafting of the manuscript. HP: critical revision of the manuscript for important intellectual content. All authors contributed to the article and approved the submitted version.

## Funding

This research was supported by Postgraduate Research & Practice Innovation Program of Jiangsu Province (project no. SJCX20-1384).

## Conflict of Interest

The authors declare that the research was conducted in the absence of any commercial or financial relationships that could be construed as a potential conflict of interest.

## Publisher's Note

All claims expressed in this article are solely those of the authors and do not necessarily represent those of their affiliated organizations, or those of the publisher, the editors and the reviewers. Any product that may be evaluated in this article, or claim that may be made by its manufacturer, is not guaranteed or endorsed by the publisher.
